# Glycation and a Spark of ALEs (Advanced Lipoxidation End Products) – Igniting RAGE/Diaphanous-1 and Cardiometabolic Disease

**DOI:** 10.3389/fcvm.2022.937071

**Published:** 2022-06-24

**Authors:** Lakshmi Arivazhagan, Raquel López-Díez, Alexander Shekhtman, Ravichandran Ramasamy, Ann Marie Schmidt

**Affiliations:** ^1^Diabetes Research Program, Department of Medicine, New York University Grossman School of Medicine, New York, NY, United States; ^2^Department of Chemistry, The State University of New York at Albany, Albany, NY, United States

**Keywords:** RAGE axis, glycation, lipoxidation, cardiometabolic disease, obesity, non-alcoholic fatty liver disease, lipid metabolism

## Abstract

Obesity and non-alcoholic fatty liver disease (NAFLD) are on the rise world-wide; despite fervent advocacy for healthier diets and enhanced physical activity, these disorders persist unabated and, long-term, are major causes of morbidity and mortality. Numerous fundamental biochemical and molecular pathways participate in these events at incipient, mid- and advanced stages during atherogenesis and impaired regression of established atherosclerosis. It is proposed that upon the consumption of high fat/high sugar diets, the production of receptor for advanced glycation end products (RAGE) ligands, advanced glycation end products (AGEs) and advanced lipoxidation end products (ALEs), contribute to the development of foam cells, endothelial injury, vascular inflammation, and, ultimately, atherosclerosis and its consequences. RAGE/Diaphanous-1 (DIAPH1) increases macrophage foam cell formation; decreases cholesterol efflux and causes foam cells to produce and release damage associated molecular patterns (DAMPs) molecules, which are also ligands of RAGE. DAMPs stimulate upregulation of Interferon Regulatory Factor 7 (IRF7) in macrophages, which exacerbates vascular inflammation and further perturbs cholesterol metabolism. Obesity and NAFLD, characterized by the upregulation of AGEs, ALEs and DAMPs in the target tissues, contribute to insulin resistance, hyperglycemia and type two diabetes. Once in motion, a vicious cycle of RAGE ligand production and exacerbation of RAGE/DIAPH1 signaling ensues, which, if left unchecked, augments cardiometabolic disease and its consequences. This Review focuses on RAGE/DIAPH1 and its role in perturbation of metabolism and processes that converge to augur cardiovascular disease.

## Introduction

The receptor for advanced glycation end products (RAGE), a member of the immunoglobulin (Ig) superfamily of cell surface molecules, was discovered on account of its ability to bind the advanced glycation end products, or AGEs. The extracellular domains of RAGE are composed of one Variable (V)-type Ig domain followed by two Constant (C)-type Ig domains (1, 2). The key to understanding the breadth of RAGE activities emerged from multiple investigations that highlighted roles for RAGE as a multi-ligand receptor. In addition to the AGEs ligands, RAGE binds multiple members of the S100/calgranulin family; amphoterin (also called high mobility group box 1 or HMGB1); amyloid beta peptide; lysophosphatidic acid (LPA); phosphatidylserine (PS); and Mac-1, as examples (1, 2). The cytoplasmic tail of RAGE binds to the formin, Diaphanous-1 (DIAPH1) (3), and this interaction was shown in cultured cells to be important for RAGE signaling. Specifically, when the key amino acids in the RAGE cytoplasmic tail responsible for binding to DIAPH1 were mutated (R5/Q6), which correspond to R366/Q367 in the full-length RAGE, to alanine residues, binding to DIAPH1 and ligand-induced cellular signaling were reduced (4).

Beyond these discoveries, RAGE also binds ligands relevant to lipid moieties and evidence is mounting that the RAGE axis regulates transcriptional programs that significantly affect lipid metabolism. This Review will consider these lipid-associated ligands of RAGE and how these ligand families, through RAGE, are importantly involved in the pathogenesis of atherosclerosis, obesity and associated liver diseases.

## Lipid-Associated Ligands of Rage

### Advanced Lipoxidation End Products

In addition to the classical sugar-mediated modifications that yield the AGEs, advanced lipoxidation end products (ALEs) also bind to RAGE. ALEs are produced through the reaction of amino acids (such as lysine) with lipoxidation products; examples of which include 4-hydroxy-trans-2-nonenal (HNE), acrolein (ACR) and malondialdehyde (MDA) (5). These species were recently shown to bind to RAGE particularly through the RAGE extracellular VC1 domain structural unit (5, 6). A chief signal transduction-stimulating ligand of RAGE, carboxymethyllysine or CML-AGE (7), may be formed both by glycoxidation and lipoxidation reactions (8). A unifying mechanism by which AGE/ALE mediates cellular stress through RAGE is *via* the generation of reactive oxygen species (ROS) (9).

It is important to note that ALEs, just like AGEs, interact with distinct receptors besides RAGE. For example, in the kidney, where ALE interaction with Galectin-3 is important for the uptake and removal of ALEs, in contrast, upon ALE interaction with RAGE, ALEs activate pro-inflammatory pathways that are linked to the development of fibrotic and inflammatory injuries in the kidney tissue (10). In addition to ALEs, other studies have shown that RAGE binds distinct lipid-related species.

### Lysophosphatidic Acid

LPA is a circulating phospholipid that acts through classical G-protein coupled receptors (GPCRs) to mediate a range of biological responses in diverse cell types (11); LPA also binds to RAGE. Through surface plasmon resonance (SPR) and nuclear magnetic resonance (NMR) studies, LPA was found to bind to the V-type Ig domain of RAGE (12). In vascular smooth muscle cells (SMCs), incubation with LPA resulted in activation of signal transduction pathways.

It was previously shown that LPA enhances the implantation and metastasis of RAGE-expressing ID8 cells, a murine epithelial ovarian cancer cell line (13). *In vivo*, upon injection of ID8 cells into C57BL/6 mice, LPA is required for the development of tumor growth and metastasis. In LPA-treated mice injected with ID8 cells, treatment with soluble (s) RAGE or performance of these studies in *Ager* null vs. wild-type mice resulted in a significant reduction in tumor cell burden and metastasis (12). It is notable that tumors were not fully eliminated in these sRAGE-treated or *Ager* null mice, consistent with the expression of non-RAGE receptors for LPA on these tumor cells.

It is noteworthy that distinct studies implicated LPA-RAGE interaction in lung, mammary and ovarian tumors (14, 15). Other studies suggested roles for LPA-RAGE in stroke (16) and upon exposure to World Trade Center particulate matter, the released LPA induced inflammation in macrophages, at least in part through RAGE (17).

### Phosphatidylserine

Insights into potentially homeostatic roles for RAGE were uncovered through studies reporting that RAGE bound the glycerophospholipid, phosphatidylserine (PS), which plays key roles in the process of apoptosis (18). In binding experiments, by surface plasmon resonance (SPR), RAGE bound PS (K_D_, 563 nM) and demonstrated very slight binding to phosphatidylglycerol. No binding of RAGE was demonstrated in the cases of diacylglycerol (DAG), phosphatidic acid (PA), phosphatidylinositol (PI), phosphatidylethanolamine (PE), phosphatidylcholine (PC) or sphingomyelin (SM) (18). Using confocal microscopy imaging and fluorescence resonance energy transfer (FRET) techniques, it was shown that RAGE co-localized with PS on apoptotic thymocytes, at least in part in pseudopod structures (18). *In vivo* experiments in mouse models supported the relevance of RAGE-PS binding, as both sRAGE and deficiency of *Ager* in mice were shown to impair macrophage phagocytosis (18). Although it is notable in these studies that the biophysical basis of RAGE binding selectively to PS was not probed; that is, why RAGE did not bind to PE, PC, or PA. For example, the results provide insight into the degree of meticulous specificity of RAGE-dependent processes in the context of lipid-stimulated biological signaling mechanisms.

Collectively, these studies describing examples of lipid-related ligands of RAGE, such as the ALEs, LPA and PS opened the doors to understanding that non-peptide/non-protein biochemical species were able to bind to RAGE. Given the vast roles for lipids as key components of cell membranes and as critical components of signal transduction rafts, and their fundamental roles in cholesterol and triglyceride metabolism with implications for human obesity and cardiovascular diseases, as examples, these findings define important roles for RAGE in human pathobiologies. In the section to follow, the links between RAGE and foam cell biology will be considered.

## Foam Cell Formation: Implications for the Rage Pathway

### Modified Lipids, Foam Cell Formation and the Effects of Diabetes

In the process of atherogenesis, apolipoprotein B (ApoB)-containing lipoproteins, such as low-density lipoprotein (LDL), and the remnant lipoproteins including very low-density lipoprotein (VLDL), intermediate density lipoprotein (IDL) and chylomicron remnants, may traverse the endothelial cell (EC) barrier into the vascular intima, and, especially in sites of dense extracellular matrix (ECM), these particles may become trapped (19, 20). Biochemical processes in this microenvironment significantly affect the nature of these lipids, such as oxidation, proteolysis, lipolysis and inflammation, as examples (21). Once formed, these lipid-modified species may be recognized and taken up by macrophages, leading to the production of foam cells; processes that begin early in life (22). It is also reported that smooth muscle cells also host these modified lipids and contribute to the pool of foam cells (23).

In diabetes, additional biochemical processes are spurred through the presence of increased concentrations of glucose, leading to activation of the Maillard reaction and the production of glycation-modified lipids (24). The principal amino acid residue in Apo-B 100 that undergoes glycation is lysine; lysine residues are important for recognition by the LDL receptor (LDLR); hence, it is not surprising that glycation promotes an increase in the half-life of glycated LDL in human diabetic vs. non-diabetic plasma (25). Additional pathways that modify lipids and are relevant in the diabetic environment include the effects of carbonyl stress, which lead to the production of ALEs, which were reviewed above and noted as ligands of RAGE; and through increased inflammatory processes observed in the vessel wall and mediated through myeloperoxidase and additional oxidative stress programs (24).

In the sections to follow, the links between lipid modifications, foam cells and the components of the AGE-RAGE pathway are considered.

### AGE Receptors and Foam Cell Biology

The first clues suggesting that the RAGE pathway might play roles in foam cell formation emerged from a series of studies reporting that in addition to RAGE, the ligand AGEs also bound scavenger type receptors known to play roles in uptake of oxidized (ox) LDL. For example, CD36 binds both AGEs and oxLDL (26). Other studies reported that scavenger receptors Scavenger Receptor A (SR-A), SR-B1 and Lectin like oxLDL receptor (LOX-1) for oxLDL also bound AGEs (27). A number of studies reported that AGEs, at least in part through RAGE, increased macrophage uptake of oxLDL and increased the levels of macrophage cholesterol esters, leading to the formation of lipid-laden macrophages, also called “foam cells” (28).

### RAGE/DIAPH1 and Foam Cell Formation

Numerous studies have implicated the ligand-RAGE pathway to foam cell formation and have identified putative mediating downstream pathways. AGEs were shown to mediate uptake of oxLDL through upregulation of *Cdk5* (cyclin dependent kinase 5) and *Cd36* in macrophages. These mechanisms required AGE-RAGE-mediated generation of oxidative stress, as inhibition of oxidative stress using N-acetyl-cysteine (NAC) and RAGE aptamers blocked AGE-mediated upregulation of *Cdk5* and *Cd36*, and, consequently blocked AGE-mediated foam cell formation (29). Other studies implicated the RAGE ligand S100A12 (30) in foam cell formation at least in part through CD36, RAGE and toll like receptor 4 (TLR4)-dependent mechanisms (31). Furthermore, AGE-RAGE-dependent roles in smooth muscle cells (SMCs) were also implicated in foam cell formation as a specific AGE, CML-AGE stimulated SMC lipid uptake and foam cell formation, along with the transdifferentiation of these cells into macrophage-like cells, at least in part via RAGE (32).

Roles for RAGE and DIAPH1 in these processes were demonstrated through studies using a new class of RAGE-DIAPH1 antagonists (33, 34). Specifically, these small molecules bind to the cytoplasmic tail of RAGE and block the RAGE tail interaction with DIAPH1; this has been illustrated directly using *in vitro* binding assays as well as FRET assays (34). A representative small molecule antagonist of RAGE/DIAPH1 was shown to suppress uptake of oxLDL and foam cell formation in wild-type RAGE-expressing macrophages (35). In parallel, it was shown that in macrophages, the small molecule RAGE/DIAPH1 antagonist blocked AGE-mediated RAC1 activity, activation of NF-kB, production of proinflammatory cytokines and cellular migration (35).

In addition to roles for RAGE in foam cell formation, evidence indicates that the RAGE pathway contributes to reduction in both macrophage cholesterol efflux and to *in vivo* reverse cholesterol transport. These processes together with ligand-RAGE-dependent increased foam cell formation, may synergize to contribute to atherosclerosis. In the sections to follow, roles for RAGE in efflux of cholesterol are considered.

## Macrophage Cholesterol Efflux and Reverse Cholesterol Transport and the Rage Pathway

Evidence from human subjects suggests that one of the mechanisms by which diabetes may accelerate atherosclerosis is through suppression of serum cholesterol efflux capacity and impaired reverse cholesterol transport (36, 37). These findings have been recapitulated in animal models of type 1 diabetes in which it was shown that macrophage-to-feces reverse cholesterol transport was impaired (38). Support for roles for RAGE and its ligands in these processes emerged from multiple *in vitro* and *in vivo* analyses.

The first reports linking RAGE to cholesterol efflux pathways were highlighted in human primary macrophages in which *in vitro*-prepared AGE albumin was shown to reduce mRNA and protein expression of ABCG1 (ATP binding cassette transporter G1), but not ABCA1 (ATP binding cassette transporter A1) (39). Roles for RAGE were established through the suppressive effects of anti-RAGE antibodies on AGE albumin functions. It was also noted in that study that at the functional level, AGE albumin reduced macrophage cholesterol efflux to High Density Lipoprotein (HDL) but not to Apolipoprotein A1 (ApoA1). Interestingly, that work showed that the effects of AGE albumin were independent of the LXR (Liver X Receptor) pathway (39).

Subsequent studies employed bone marrow -derived macrophages (BMDMs) from wild-type or *Ager* (the gene encoding RAGE)-deficient mice and showed that macrophage cholesterol efflux to both HDL and ApoA1 was suppressed upon *Ager* deletion, in parallel with reduced levels of *Abcg1* and *Abca1* mRNA and ABCG1 and ABCA1 protein (40). In *in vitro* studies using human THP1 macrophage like cells, the effects of RAGE on regulation of these transporters were traced to peroxisome proliferator activated receptor-gamma (PPAR-gamma)-dependent mechanisms and not to LXR pathways (40). Using macrophages from diabetic wild-type or *Ager* null mice, it was shown that macrophage reverse cholesterol transport to plasma, liver and feces was reduced in diabetic mice *via* RAGE (40).

Beyond *in vitro*-prepared AGEs, isolated AGE albumin from human type 1 or type 2 diabetic plasma vs. control non-diabetic plasma was also demonstrated to reduce macrophage cholesterol efflux to human THP1 cells or to wild type BMDMs *via* RAGE (41). In that study, human diabetic AGE albumin regulated macrophage inflammatory and pro-oxidative gene expression *via* RAGE (41). Beyond AGEs, other RAGE ligands were also shown to reduce macrophage cholesterol efflux; for example, S100B, S100A8/A9 and S100A12 also played roles in these processes (42, 43).

Finally, it has also been suggested that statin drugs may act, in part, by reducing AGE-mediated suppression of cholesterol efflux. For example, rosuvastatin blocks AGE-mediated reduction in macrophage cholesterol efflux through blockade of AGE-induced oxidative stress (44). In other studies, in THP1 macrophages, treatment with atorvastatin increased cholesterol efflux and expression of the transporter ABCG1, phenomena which were found to be reduced by AGEs treatment. In addition, treatment with atorvastatin reduced RAGE expression in macrophages as well (45).

Collectively, these findings using mouse and human cells as well as mice and human subject-derived AGE albumin implicate the RAGE pathway in key macrophage properties that contribute to foam cell formation and failure of resolution in atherosclerosis especially in diabetes but in the non-diabetic state as well. In this context, recent research has uncovered new mechanisms by which RAGE/DIAPH1 might contribute to regulation of cholesterol metabolism through the regulation of Interferon Regulatory Factor 7 (IRF7).

## The Rage Pathway, Atherosclerosis and Regulation of IRF7

Although extensive reports implicated RAGE in the development/progression of atherosclerosis (non-diabetes and diabetes) in mouse models (46–59), little was known about potential roles for RAGE/DIAPH1 in the regression of atherosclerosis, particularly in diabetes.

To test roles for RAGE in regression of diabetic atherosclerosis, a murine model of aortic arch transplantation was performed. In that model, mice devoid of the *Ldlr* were rendered diabetic with streptozotocin and then fed a Western diet. Upon development of atherosclerosis, aortic arches were surgically interpositioned into the vasculature of the recipient diabetic wild-type, *Ager* null or *Diaph1* null mice (60). Compared to wild-type diabetic recipient mice, diabetic mice devoid of *Ager* or *Diaph1* displayed accelerated regression of atherosclerosis with smaller atherosclerotic lesion areas, reduced lesional neutral lipid staining (Oil Red O) and reduced macrophage content per lesion area (CD68^+^ cells).

In order to identify the underlying mechanisms, donor diabetic *Ldlr* null aortic arches (CD45.2) were transplanted into the above recipient mice (CD45.1) (60). At five days after transplantation, flow cytometry experiments revealed that there were no differences in the percent recipient CD45.1 vs. donor CD45.2 of the CD11B^+^/F4/80^+^ macrophage lesional content by recipient genotype (wild-type vs. *Ager* null), but that there were trends to higher recipient CD45.1/CD68^+^ macrophage content in the aortic arch lesions transplanted into the *Ager* null vs. the WT diabetic recipient mice. Overall, these findings were consistent with earlier work that demonstrated that the majority of the aortic arch lesional macrophages during the regression process are accounted for by newly recruited cells from the recipient mice (60, 61) and it was further demonstrated that expression or deficiency of *Ager* did not affect the relative contributions of donor vs. recipient macrophages in the regression environment.

On account of the ability to discern donor CD45.2 vs. recipient macrophages CD45.1 in the atherosclerotic plaques in this experimental paradigm, bulk RNA-sequencing was performed on sorted macrophages from the distinct groups of diabetic mice. Bioinformatics analysis revealed downregulation of three pathways in the *Ager* null vs. wild-type recipient macrophages that were linked to the interferon signaling, specifically interferon alpha/beta signaling and gamma signaling (60). In contrast, the fourth pathway identified by this analysis revealed a significant increase in “glycolysis” pathway in *Ager* null vs. wild-type recipient macrophages. Further analysis of the differentially-expressed genes uncovered that *Irf7* mRNA was significantly lower in the *Ager* null vs. wild-type recipient CD45.1 macrophages.

In *in vitro* studies, BMDMs were treated with serum from Western diet–fed Ldlr null mice, which is enriched in RAGE ligand DAMPs, or with isolated RAGE ligand CML-AGE; compared to wild-type BMDMs, those devoid of *Ager* demonstrated lower levels of Irf7 mRNA in the presence of these RAGE ligands. Through these studies, roles for *Irf7* in regulation of cholesterol metabolism and inflammation in macrophages were also identified. BMDMs were grown in the serum from Western diet-fed mice devoid of the *Ldlr*. Compared with scrambled siRNA, Irf7 knockdown resulted in significant upregulation of genes linked to cholesterol efflux (Abca1 and Abcg1), and increased expression of Nr1h2 (LXRβ), Nr1h3 (LXRα), and Srebp1 and Scap. Significant reductions in Cd36 were observed in Irf7-knockdown vs. scramble siRNA–treated cells as well. In parallel, BMDMs exposed to Western diet–fed Ldlr null mice serum in the presence of Irf7 knockdown displayed lower levels of cholesterol. Finally, compared with scrambled siRNA, Irf7 knockdown resulted in upregulation of Arg1 and Il10 and reduction in Tnfa, Nos2, Il6, and Ccl2 (60). Collectively, these findings linked RAGE to regulation of IRF7 and uncovered intriguing roles for RAGE/IRF7 in cholesterol metabolism and inflammation. Future studies will need to address the full range and implication of these findings to the response to viral infection, atherosclerosis, and obesity, as examples of disorders in which key roles for “immunometabolism” have been suggested. Experiments designed to test these hypotheses and relationships are underway.

In this report describing roles for RAGE in atherosclerosis regression in diabetes, unexpected differences in circulating lipids were also noted. Specifically, compared to diabetic wild-type recipient mice in the aorta transplantation model, diabetic mice devoid of *Ager* or devoid of *Diaph1* demonstrated modest but significantly lower levels of total cholesterol with no differences in triglyceride levels (60). Although the mechanisms underlying these intriguing observations are yet to be discovered, they are under intensive investigation at this time.

In the final sections of this review, roles for RAGE in disrupted lipid metabolism in obesity and non-alcoholic fatty liver disease (NAFLD) have been identified and will be considered.

## Aberrant Ligand-Rage Interaction and Lipid Dysregulation: Implications for the Pathogenesis of Obesity

### RAGE Expression and Actions in Adipocytes

Although initial work upon the discovery of RAGE focused largely on the impact of its expression in vascular cells, immune cells, cardiomyocytes and neurons, as examples, ongoing investigations illustrated that RAGE was also expressed on adipocytes. In studies in 3T3-L1 adipocyte-like cells, a range of AGEs exerted maladaptive effects *via* RAGE (62). First, it was shown that AGEs derived from glucose, glyceraldehyde, or glycolaldehyde inhibited the differentiation of 3T3-L1 cells. Second, these AGEs suppressed glucose uptake in the presence or absence of insulin. These effects of AGEs were prevented by treatment of the cells with antibodies against AGEs or against RAGE. The RAGE-dependent downstream mechanisms in these pathways were traced to oxidative stress, as treatment of the 3T3-L1 adipocytes with N-acetylcysteine blocked the effects of AGEs on these metabolic consequences (62). Third, it was shown that AGE treatment of 3T3-L1 adipocytes also induced upregulation of monocyte chemoattractant protein 1 (MCP1), thereby identifying a mechanism to stimulate pro-inflammatory effects in AGE-treated adipocytes.

In other studies in a human preadipocyte cell line, SW872 cells, secretion of HMGB1 was demonstrated, which led to the production of IL6. In those cells, a direct role for HMGB1 in the production of IL6 was shown by treatment with recombinant HMGB1, which stimulated production of IL6 (63). The effects of recombinant HMGB1 on regulation of IL6 production were dependent on RAGE and not on TLR2 or TLR4. Others showed that AGE-RAGE in adipocytes was linked to outcomes such as matrix metalloproteinase (MMP) activity (64) and induction of insulin resistance through regulation of GLUT4 activities (65).

Links to DIAPH1 in regulation of adipocyte functions were also demonstrated in other recently reported work. Specifically, visceral adipocytes were retrieved from human subjects with and without diabetes and were examined in both 2-dimensional (2D) and 3D-ECM systems. These authors reported that AGE content was higher in the diabetic vs. nondiabetic adipocytes. In studying potential effects of AGEs, glycated collagen one and AGE-modified ECM materials were shown to regulate glucose uptake, and upregulate expression of *AGER* and *CD36* in the adipocytes as well as expression of RHO GTPase signaling mediators including DIAPH1 (66). Interestingly, in this system, inhibition of DIAPH1, but not RAGE or CD36, reduced AGE-ECM-mediated inhibition of glucose uptake in the adipocytes (66). Although not directly tested in that work, it is possible that the direct links between DIAPH1 and actin cytoskeleton dynamics were responsible for DIAPH1's impact on AGE-ECM properties in adipocytes.

These concepts linking RAGE to obesity have been tested in animal models of high fat diet feeding or in genetically obese and diabetic mice, and will be considered in the sections to follow.

### RAGE in Murine Models of Obesity

Numerous reports have tested the role of RAGE in mice fed a high fat diet (60% fat content). Mice bearing global or bone marrow-specific deletion of *Ager* displayed significant protection from diet-induced obesity and adiposity (67). In parallel, mice globally devoid of *Ager* and fed a high fat diet also displayed improved glucose and insulin tolerance. Interestingly, these studies showed that mice bearing global deletion of *Ager* demonstrated higher energy expenditure compared to wild-type mice fed the high fat diet. To more specifically probe the role of RAGE in energy expenditure, mice bearing adipocyte-specific deletion of *Ager* were studied in settings that would evoke a definitive metabolic response. Compared to the wild-type *Ager*-bearing control animals, mice devoid of adipocyte *Ager* demonstrated superior metabolic recovery after fasting, a 4°C cold challenge, or high-fat feeding. Using both *in vivo* and *in vitro* methodologies, that work illustrated that RAGE-dependent mechanisms included suppression of protein kinase A (PKA)-mediated phosphorylation of its major targets, hormone-sensitive lipase (HSL) and p38 mitogen-activated protein (MAP) kinase, upon β-adrenergic receptor stimulation (68) and that these processes reduced the expression and activity of uncoupling protein 1 (UCP1) and, therefore, the associated thermogenic programs (68).

It is important to note that a distinct publication suggested that mice devoid of *Ager* displayed increased weight gain on a high fat diet (60% kcal fat) vs. *Ager*-expressing control mice (69). Although it is not possible to discern the reasons for the varied findings between the studies, it is possible that the genetic background of the mice, the mode of breeding of the mice, as well as other factors within the environment and the health status of the vivarium, may have differed between the studies. Furthermore, it is notable that beyond global deletion of *Ager*, the studies described above also employed bone marrow or adipocyte-specific deletion of *Ager* (in which protection from diet-induced obesity was shown). In that work, treatment of wild-type C57BL/6 mice with soluble RAGE who were fed a high fat diet also demonstrated significant reductions in gain of body mass vs. the vehicle-treated controls (67).

Studies in human subjects are being conducted to accrue correlations between the RAGE pathway and human obesity and overweight states. Relationships between RAGE ligands and RAGE expression in adipocytes and other cell types relevant to metabolic dysfunction suggest that it is logical to test contributory and mediating roles for this pathway in human obesity.

### RAGE/DIAPH1 and Human Obesity

A typical means by which RAGE is assessed *in vivo* in human subjects is through the measurement of sRAGE; total sRAGE is composed of both a cell surface cleaved form of the receptor, which represents about 80% of total sRAGE and the soluble product of an mRNA splice variant of *AGER* that results in production of endogenous secretory or esRAGE (70). There are multiple examples of clinical settings relevant to obesity in which sRAGEs were measured. First, studies compared levels of sRAGE in patients with obesity vs. healthy controls and found that sRAGE levels were lower in patients with obesity vs. healthy weight persons (71). After bariatric surgery and weight loss, it was found that the levels of sRAGE rose. Correlation models were performed and yielded the following insights: the differences in levels of sRAGE were associated with the differences in 1 and 2 h post-prandial glucose, differences in fasting insulin, differences in 2 h post-prandial insulin levels, differences in homeostatic model assessment-insulin resistance (HOMA-IR) model and the differences in levels of triglyceride. In the multivariate model, the differences in 1 and 2 h post-prandial glucose, the differences in 2 h post-prandial insulin and differences in HOMA-IR predicted the differences in sRAGE. Hence, these studies suggested that the differences in levels of sRAGE were coupled to measures of glucose and insulin tolerance. Other studies in patients undergoing bariatric surgery also queried if differences in levels of sRAGE were observed. Patients with obesity were randomized to either bariatric surgery or medical weight loss and levels of sRAGE were observed pre-surgery and at 6 months post-surgery. In the patients undergoing bariatric surgery, but not in the medical weight loss group, higher baseline levels of sRAGE were associated with better weight loss outcomes (72).

Distinct studies also examined the levels of RAGE ligands, particularly CML-AGE in obese adipose tissues. In human obese vs. lean adipose tissue, even in the absence of diabetes, higher levels of CML-AGE and RAGE expression were observed (73). To explain the lower plasma levels of CML-AGE in patients with obesity vs. lean state, a model was developed in mice with genetically mediated diabetes (*db/db* mice); the results of studies in this model suggested that plasma levels of CML-AGE were lower in obesity on account of trapping of the RAGE ligand in the obese (high RAGE-expressing) adipose tissue. Furthermore, upon more in-depth analyses, it was shown that the decreased plasma levels of CML-AGE in patients with obesity were strongly associated with insulin resistance (73).

Studies reported from the Northern Manhattan Study (NOMAS) examined a diverse group of human subjects and reported that median sRAGE levels were significantly lower in subjects who had elevated waist circumference, blood pressure, and fasting glucose, but that no relationship was observed between levels of sRAGE and elevated triglycerides or reduced HDL levels that were also noted in these subjects (74). Strikingly, when the NOMAS investigators performed additional stratification and interaction analyses, they uncovered the key finding that the association of sRAGE levels with metabolic syndrome factors was more prominent in Hispanic vs. White persons and that there was no association with the components of the metabolic syndrome in Black persons (74). These results thus indicate that more research is required in order to determine potential mechanisms underlying these findings, and if racial background should be considered in the interpretation of studies tracking sRAGE levels in cardiometabolic diseases and therapeutic approaches.

Finally, recent work has addressed the potential relationships between *AGER* and *DIAPH1* expression and metabolic health in patients with obesity. The mRNA expression of these genes and multiple other genes involved in glycation and inflammation was studied in human omental and subcutaneous adipose tissue in patients with obesity. The key findings from that study further cemented the relationship between *AGER* and *DIAPH1*; it was reported that only in subcutaneous but not omental adipose tissues, the mRNA expression of *AGER* significantly correlated with that of *DIAPH1* (75). Only in subcutaneous but not omental adipose tissue, genes linked to the AGE-RAGE-DIAPH1 axis strongly correlated with markers of inflammation and adipogenesis and, intriguingly, in subcutaneous but not omental adipose tissue, the mRNA expression of *AGER* was found to correlate significantly with HOMA-IR (75). Whereas, some of these associations, particularly to inflammation markers and HOMA-IR might have been expected to be uncovered between *AGER* and *DIAPH1* in omental adipose tissue, this was not the case. These interesting findings pinpoint future directions for more in-depth study of RAGE/DIAPH1 biology in the subcutaneous adipose tissue depot. As non-alcoholic fatty liver disease (NAFLD) commonly accompanies obesity and is associated with excess lipid content in the liver, potential roles for the RAGE pathway in this setting have been tested as well and will be reviewed in the section to follow.

## The Rage Pathway and NAFLD

Obesity is closely associated with the development of NAFLD and non-alcoholic steatohepatitis (NASH); a recent Statement published from the American Heart Association indicated that the majority of patients presenting with NAFLD also suffer with obesity, as no more than 20% of NAFLD cases in the United States and Europe were observed in lean persons (76, 77).

Associations between the RAGE pathway and NAFLD have been suggested by multiple studies showing the hepatic and plasma/serum levels of RAGE ligands AGEs, ALEs, S100 and HMGB1, were enhanced in NAFLD vs. healthy controls (78–82). RAGE is expressed on multiple cell types in the liver, such as hepatocytes, stellate cells, Kupffer cells, infiltrating immune cells and vascular cells (83, 84). Interestingly, in conditions in which NAFLD-type disorders occur, either through diet or genetic modification, global deletion of *Ager* is not protective against steatosis, inflammation or fibrosis (85, 86). In contrast, mice bearing deletion of hepatocyte *Ager* and fed a high-AGE diet were protected from hepatic inflammation and fibrosis through suppression of the adverse effects of RAGE-induced oxidative stress (87). In other studies, reduction of *Ager* expression by adenoviruses injection reduced hepatosteatosis and liver inflammation in aged mice (88). Thus, these studies, employing various diets, genetic models and distinct means to silence *Ager* in a NAFLD-like environment, highlight that RAGE may play adaptive vs. deleterious roles in NAFLD-like conditions, and that more research is needed to dissect cell-intrinsic and cell-cell cross-talk mechanisms in hepatic metabolic dysfunction.

Studies are underway to probe how DIAPH1 may contribute to hepatic lipid metabolism as well given its relationship to RAGE signal transduction. In first studies in mice with a type 1-like diabetes induced by streptozotocin and fed normal rodent chow, ^1^H magnetic resonance spectroscopy and chemical shift-encoded magnetic resonance imaging techniques revealed that compared to wild-type mice, mice globally devoid of *Diaph1* displayed significant reductions in hepatic and cardiac triglyceride content (89). These findings suggest that DIAPH1 contributes to lipid metabolism and potential roles for RAGE in these processes and the precise mediating mechanisms are under intensive investigation.

## Summary

**Figure 1:**
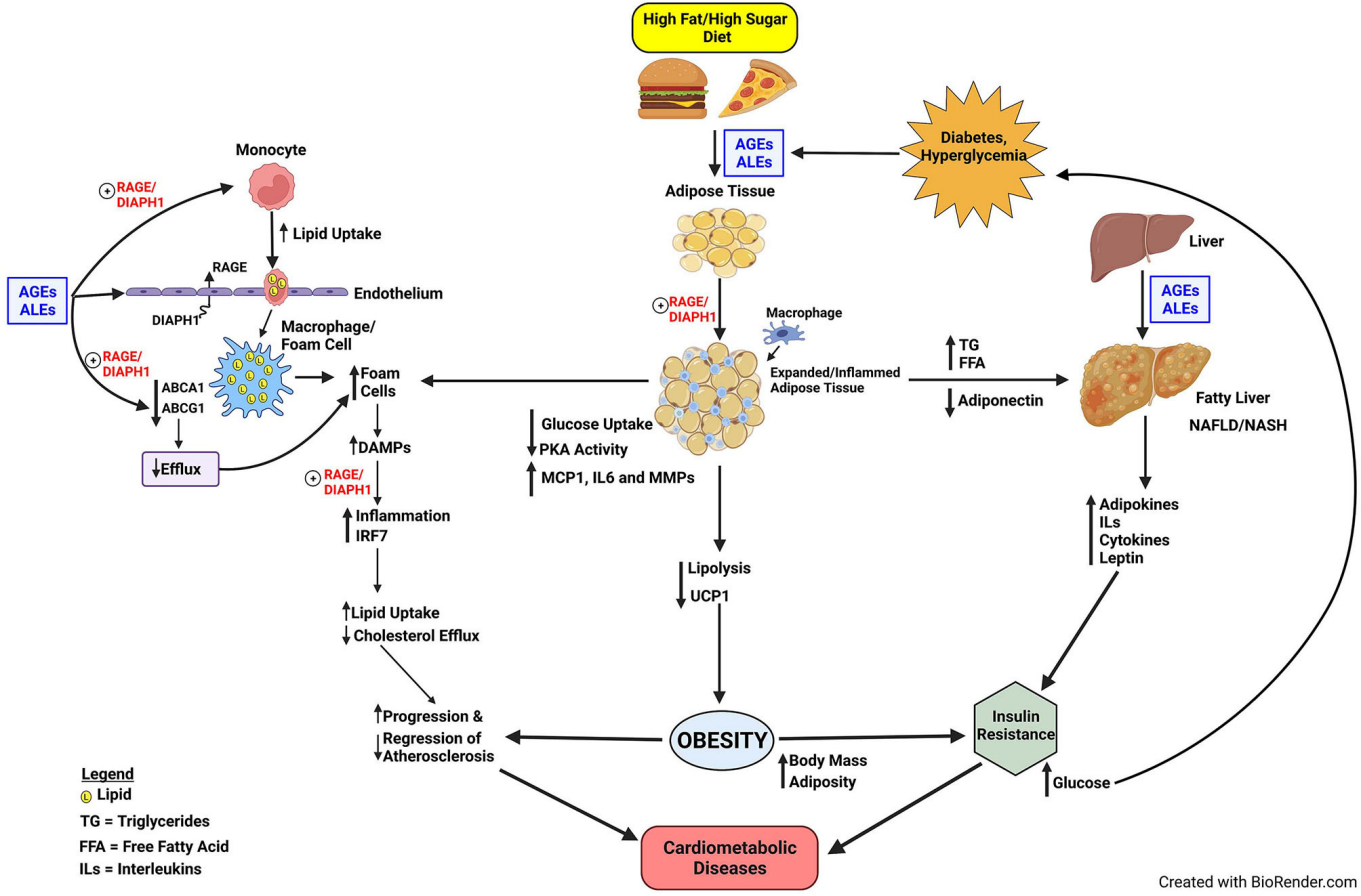
Proposed model of RAGE/DIAPH1 in glycation and lipid metabolism and implications for cardiometabolic disease. Diets high in fats and sugars trigger the production of AGEs and ALEs, which are ligands of RAGE. In very early events in atherogenesis, the production of foam cells in monocytes/macrophages and smooth muscle cells may be facilitated by AGE/ALE signaling through RAGE/DIAPH1. Foam cells, characterized by heightened inflammation in the atherosclerotic vessel wall, produce RAGE ligand DAMPs, which perpetuate inflammatory responses *via* RAGE/DIAPH1; one consequence of which is upregulation of IRF7, which itself perturbs inflammation and cholesterol metabolism pathways and processes that exacerbate progression of atherosclerosis and delay its regression. AGEs and ALEs also accumulate in obese adipose tissue and liver, the latter especially relevant to the pathogenesis of NAFLD. Obesity and NAFLD contribute to the development of insulin resistance, and, therefore, the potential for development of hyperglycemia and processes that amplify AGE/ALE production and their vicious cycle of cellular stress. The optimal means to divert RAGE-DIAPH1 binding and downstream signaling is under intensive investigation and is hypothesized to reduce cardiometabolic risk and its consequences.

Disorders of glucose and lipid metabolism are central to the pathogenesis of cardiometabolic diseases and the increased risk for obesity, NAFLD/NASH, atherosclerosis, heart attacks, strokes, and peripheral vascular diseases; processes which may culminate in severe complications such as heart failure, irreversible neurological damage and dementia, and amputations of digits and limbs. Although the RAGE axis was discovered on account of the ability of the AGEs to bind and signal *via* this receptor, soon after its identification, its role in transducing the signals triggered by ALEs expanded the implications of RAGE pathobiologies to those mediated by pathological lipid species. Furthermore, the discoveries that RAGE bound non-AGE and non-ALE ligands expanded our understanding of the biology of RAGE and implicated this receptor in inflammation, lipid signaling and activation of the innate immune system (Figure 1).

Well beyond the implications for roles in the pathological consequences of sugar-modified molecules, the above cited studies recount how the RAGE pathway contributes in multiple cell types, tissues and organs to disordered lipid metabolism. In foam cells, the RAGE pathway imbues multiple hits to these cells, favoring their overall accumulation of lipids through increased uptake and decreased efflux mechanisms. The results of studies in foam cells highlighted that the RAGE pathway regulates seminal transcriptional events, and in some cases, in unique ways. For example, although the regulation of the expression of cholesterol transporters has been importantly ascribed to Liver X Receptor (LXR) pathways, RAGE-dependent downregulation of *ABCA1* and *ABCG1* was LXR-independent, and accounted for, at least in part, by PPARG.

The surprising discovery that RAGE ligands also regulated expression of IRF7 has opened new doors to probing if RAGE plays important roles in response to viral infections and the understanding that RAGE is a receptor for multiple classes of DAMPs emitted from the cytoplasm or damaged mitochondria. IRF7 has been described as a master regulator of the type 1 interferon response (90). It is fascinating that like RAGE, IRF7 signaling is also associated with MyD88 and NF-kB signaling, triggered in part by mediators such as TLR9 (91), which is also a RAGE ligand (92, 93). These discoveries support intriguing associations through which the RAGE pathway intersects with COVID19 and its important risk factors, such as obesity, diabetes and advanced aging – all conditions in which AGEs, ALEs and DAMPs are exuberantly produced (94–98).

Furthermore, although the RAGE pathway was long implicated in diabetes and diabetic complications, the RAGE ligands AGEs and ALEs accumulate in obese non-diabetic adipose tissues and may be “trapped” in the tissue by higher levels of RAGE expression, when compared to adipose tissue from lean non-diabetic subjects (73). Unanticipated roles for RAGE ligands in regulation of adipocyte glucose uptake; inflammation; and PKA activities, which mediate lipolysis and regulation of UCP1 and thermogenesis, have broadened our understanding of RAGE and suggested perhaps natural functions of the receptor. Specifically, is it plausible that RAGE functions in an energy conservation pathway to regulate PKA activities in adipocytes; in time of starvation, RAGE acts as a barometer to gauge the need for and regulate energy expenditure? However, in states of overnutrition, retention of these RAGE innate functions supports obesity and its metabolic sequelae. Similarly, in diabetes and obesity, perturbation to the liver may trigger the production of NAFLD and its implications for raising cardiometabolic risk (76). Although roles for RAGE in NAFLD may be more complex and involve both adaptive and maladaptive cellular consequences, it is plausible that prevention and treatment of the underlying causes, such as obesity, may assuage NAFLD-like conditions and reduce cardiovascular risk (Figure 1).

The observations that RAGE plays roles in diet-induced obesity and NAFLD have important implications for the development of insulin and glucose intolerance, thereby bringing roles for the RAGE pathway in cardiometabolic dysfunction to full circle. Through insulin resistance, hyperglycemia and oxidative stress may ensue, thereby triggering production of new AGEs/ALEs, more cellular stress, thereby begetting more DAMPs, and, thus, sustaining and reinforcing the cycle proposed in Figure 1.

Is this RAGE-dependent cycle of cardiometabolic stress interruptible? Numerous studies have underscored that the RAGE ligands may bind on multiple and discrete domains of the extracellular V, C1, and C2-type Ig domains (99, 100). On account of these findings by multiple research groups, it is plausible that targeting the RAGE-DIAPH1 interaction may represent a superior approach to capture the signaling impact of the multiple RAGE ligands through blocking downstream events; in this context, administration of the chemical probe, RAGE229, which binds to the cytoplasmic domain of RAGE and blocks interaction with DIAPH1, to mice blocks delayed type hypersensitivity inflammation, cardiac ischemia (diabetic mice), type 1 and type 2-diabetes like kidney disease and improves wound healing in mice with type 2-like diabetes (33, 34). Time will tell if stopping the cycle of RAGE/DIAPH1-dependent dysregulation of glucose and lipid metabolism, through this small molecule approach, may be translated to benefit for human subjects.

## Author Contributions

AMS, LA, RL-D, AS, and RR contributed to the writing of the manuscript and edited the final version. AMS wrote the first draft and edited the final manuscript. All authors contributed to the article and approved the submitted version.

## Funding

This research was funded by U.S. Public Health Service: 5P01HL146367, 1R24DK103032, 1R01DK122456-01A1, 1P01HL131481-01A1, 5R01HL132516, and 5R01DK109675. U.S. Department of Defense: W81XWH-17-1-0201 and W81XWH-17-1-0202. Support was also provided from the Diabetes Research Program, NYU Grossman School of Medicine.

## Conflict of Interest

RR, AS, and AMS have patents and patent applications through NYU Grossman School of Medicine that have been submitted/published that are related to some of the work reviewed in this manuscript. The remaining authors declare that the research was conducted in the absence of any commercial or financial relationships that could be construed as a potential conflict of interest.

## Publisher's Note

All claims expressed in this article are solely those of the authors and do not necessarily represent those of their affiliated organizations, or those of the publisher, the editors and the reviewers. Any product that may be evaluated in this article, or claim that may be made by its manufacturer, is not guaranteed or endorsed by the publisher.
